# Stumped by the Mystery: A Case Report of Progressive Shortening of Bone Following an Above-Knee Amputation

**DOI:** 10.7759/cureus.29222

**Published:** 2022-09-16

**Authors:** Freideriki Poutoglidou, Rahul Khan, Matija Krkovic

**Affiliations:** 1 Major Trauma Unit, Department of Trauma and Orthopaedics, Addenbrooke's Hospital, Cambridge University Hospitals NHS Foundation Trust, Cambridge, GBR; 2 Medical School, School of Clinical Medicine, University of Cambridge, Cambridge, GBR

**Keywords:** stump length, bone resorption, bone shortening, amputation stump, lower limb amputation

## Abstract

Several studies have investigated the anatomical adaptations in amputation stumps. In this study, we present a case report of a patient who underwent an above-the-knee amputation and, over the course of time, the length of the residual bone spontaneously shortened. The patient had undergone a total hip replacement in the same leg, and the cement mantle of the hip replacement, which could be seen within the medullary canal in the early postoperative X-rays, protruded due to bone resorption one year after the amputation*.* Although changes in bone microarchitecture in amputation stumps are well established, this is the first report of macroscopic changes in its actual length.

## Introduction

The incidence rate of major lower extremity amputation in the United Kingdom is estimated at around 5.1 cases per 100.000 population [1]. One of the main factors that determine the functional outcome after lower limb amputation is the level of bone section. It is well known that below-the-knee amputations (BKA) are associated with significantly better outcomes compared to above-the-knee amputations (AKA) [2]. The optimal stump length for a BKA is considered to be no less than 12.5cm [3]. Similarly, although consensus is lacking on the optimal length of the femur following an AKA, it has been shown that patients with longer residual limbs are able to walk faster than those with shorter residual limbs [4].

Numerous investigations have been performed regarding the anatomical adaptations in amputation stumps. It has been established that postoperative atrophy and reduced strength are seen in the muscles in the amputated limb [5]. Previous animal studies have demonstrated that shortly after amputation, stumps become hypervascularized [6, 7]. Finally, amputation stumps are characterized by marked osteoporotic changes that have been mainly attributed to insufficient mechanical load on the residual limb [8, 9].

We present a case report of a patient who underwent an AKA and, over the course of time, the length of the residual bone shortened significantly. Although the length of the residual limb is one of the main parameters that influence the functional outcome following an amputation, no investigations have previously been undertaken as to the possible alterations in the length of the bone stump.

## Case presentation

**Figure 1:**
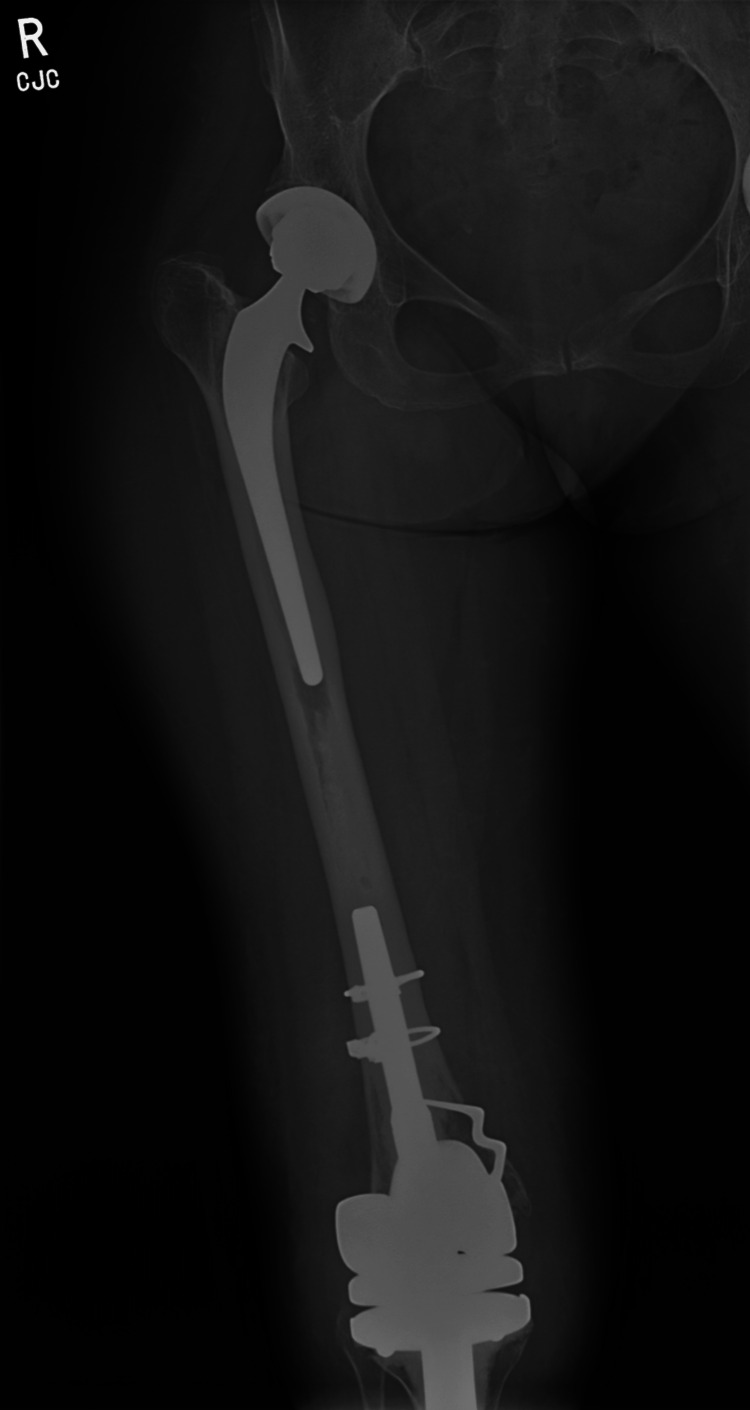
Preoperative anteroposterior X-rays of the patient showing the total hip replacement and the infected total knee replacement of her right leg

A 39-year-old female patient was referred to the Bone Infection Multidisciplinary Team of our hospital three years ago because of a chronically infected prosthetic knee joint. The patient had a history of juvenile idiopathic arthritis and no other known comorbidities. She had undergone bilateral knee replacements ten years before her referral, as well as a right total hip replacement (Figure 1). The patient presented with an associated breakdown of the skin of the right knee of 15x15 mm. The aspirate fluid from the prosthetic joint grew *Propionibacterium acnes*. Static images of the hips and knees of the patient were acquired three and twenty-four hours after injection of radiolabeled white cells. The images showed a multifocal abnormal concentration of white cells within the bone surrounding both the femoral and tibial components of the right knee. No abnormal concentration was appreciated in the region of the ipsilateral hip. A diagnosis of an infected knee replacement was established and treatment options included antibiotic suppression, a two-staged revision, or an AKA. These options and the possible complications of those were discussed with the patient and she opted for an AKA. Preoperatively, it was known that there was a continuous column of bone cement between the distal tip of the hip femoral component and the proximal tip of the knee femoral component. During the procedure, after cutting the femur at the desired point of amputation, 1.5 cm of the intramedullary cement was removed from within the canal. The rest of the cement was not removed as there was a risk of contiguous spread in the prosthetic hip joint. The postoperative recovery was uneventful and a few months later the patient completed an amputee rehabilitation program and was able to ambulate with a prosthesis.

**Figure 2:**
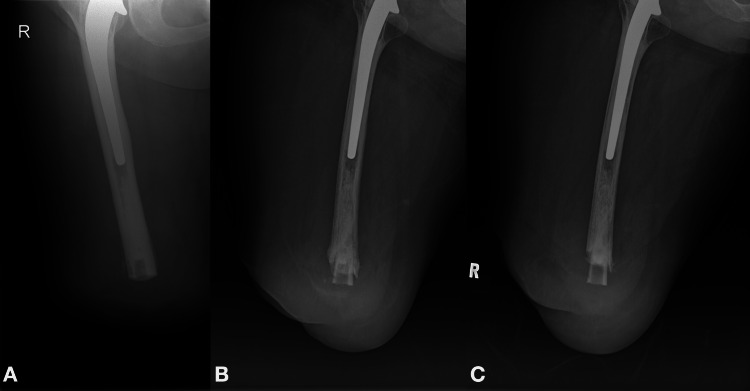
A. Anteroposterior X-ray of the amputation stump in the early postoperative period (two months postoperatively). B. Anteroposterior X-ray of the stump one year after the amputation showing absorption of the bone and shortening of the bone stump. The cement mantle protrudes from the shortened bone stump. C. Anteroposterior X-ray of the stump three years after the amputation indicating further shortening of the stump.

Due to the history of the infection, the patient was followed up yearly with X-rays of the amputation stump and inflammatory markers. Χ-rays one year after the amputation revealed absorption of the distal end of the bone that resulted in a 2 cm shortening of the bone stump. Interestingly, a tube of the outer rim of the removed cement mantle of the total hip replacement, which can be seen within the medullary canal in the early postoperative X-rays, protrudes one year later because of the bone resorption. During the last follow-up, three years after the amputation, a further shortening of the bone stump was obvious (Figure 2). Investigations showed no signs of residual infection and the patient remained ambulatory. We continue to follow the patient up closely due to concerns with regards to further resorption of the bone or recurrence of the infection.

## Discussion

Here, we present a case of a patient having undergone an AKA that was followed by progressive spontaneous shortening of the bone stump. Previous studies have demonstrated a substantial loss in bone content and strength in amputation stumps [9, 10]. However, whilst changes in bone microarchitecture are well established, this, to the best of our knowledge, is the first report of macroscopic changes of its actual length.

Bone overgrowth is a known complication after limb amputation in paediatric patients, associated with pain, skin breakdown and problems with prosthetic fit [11]. The pathogenesis of bone overgrowth in children has not been fully elucidated yet. Nevertheless, previous studies have shown that overgrowth of the stump is not a result of physeal growth but growth occurring in the medullary canal [12]. Aitken et al implanted radiographic markers in the ends of bone stumps and concluded that the overgrowth phenomenon is a result of appositional osteogenesis at the apex of the bone stump [13, 14].

The exact mechanism that led to the shortening of the bone stump in our case is not known. We hypothesize that this may be a result of excessive bone resorption due to the insufficient mechanical load on the amputated limb in addition to the history of inflammatory disease of the patient which may have resulted in some alteration in bone metabolism. Changes in the gait pattern and the lack of muscular action at the stump may also be contributing factors.

The stump shortening, in our case, has currently not been associated with any clinical manifestations. However, we will keep the patient under close review as, if resorption were to continue, further shortening could potentially cause problems with an alteration in the shape of the surrounding soft tissues. This could lead to issues with prosthetic fitting, reduced strength or even an increase in energy expenditure of walking. Future studies on the current topic are required in order to verify our findings and elucidate the etiology of this condition.

## Conclusions

This is a case report of a patient having undergone an AKA for an infected total knee replacement that was followed by progressive shortening of the bone stump leading to protrusion of the cement mantle of the pre-existing total hip replacement. Possible explanations for this may be the bone resorption due to the reduced mechanical load on the amputation stump or the history of inflammatory disease of the patient. Although shortening was not associated with any clinical symptoms, we will continue to follow up with the patient closely. Further studies could validate our findings and shed light on the underlying mechanisms that lead to bone shortening in adult amputees. 
